# A Synopsis of the Medical Botany of Illinois

**Published:** 1884-08

**Authors:** J. M. G. Carter


### ARTICLE IV.

### A SYNOPSIS OF THE MEDICAL BOTANY OF ILLINOIS. *By* J. M. G. CARTER, M. A., M. D., PH. B.

There are few States, if any, that have a more extensive list of native medical plants than Illinois. The physician has a complete materia medica growing profusely all about him wherever he goes in the Prairie State. While we are acquainted with hundreds of medicinal herbs, we are as yet only partially acquainted with their physiological and therapeutical action. One principle is beginning to dawn upon us which has often been totally ignored in studying the medical properties of plants, and even yet it is scarcely fully appreciated. That principle is this: The medical properties of the plants of a genus are identical, differing only in degree; and the properties of different genera of the same family or order are more or less related. This statement is not contradicted by the fact that remedial agents belonging to the same botanical genus are successfully used to accomplish different results. An individual property may be so marked in one plant and so feeble in another as to make the administration of these drugs produce very different effects. Thus, if A, B, C, and D are plants of the same genus which are astringent, stimulant, tonic, and diuretic in their therapeutical action, A may be the best astringent, B the best stimulant, C the best tonic, and D the best diuretic in the genus. Hence in physiological classifications if the same plants occur in separate classes, they occur in a different order of arrangement in the different lists.

Attention to the principle before given would obviate the numerous quackish laudings of new plants as specifics for diseases which were previously treated on general therapeutical principles. There is a large field for investigation yet in this department of medicine, and it would seem that great good could be accomplished if country physicians and others who have opportunity would each select a particular genus of plants and closely study their therapeutical relations and power. The heterogeneous facts so frequently given concerning medicinal agents are very confusing to a student, and not much less so to the physician. It is not possible at the present time to give an accurate physiological classification of vegetable remedies; but there is no doubt that faithful and patient study of their properties would enable us to make such a classification ere long. The field for investigation in Illinois is not less extensive than that of any other State. The following list includes all the plants of the State, so far as I am aware, that have been used in the treatment of any form of disease, either by the profession or in domestic practice. The medical properties given are those dependent upon the physiological action of the plant when that is known; but it must be admitted that the information upon this point is but too meagre. The common English names are given in parenthesis; naturalized or adventive species are written in italics.

Order Ranunculaceæ. (Crowfoot family). 11 genera, 26 species. Genus Clematis (Virgin's-Bower), C. viorna (Leatherflower); C. Virginiana (Common Virgin's-Bower). Med. prop.: Acrid, vesicant, stimulant, diuretic, sudorific.

Genus Hepatica. (Liverwort, Liverleaf). H. acutiloba (Sharplobed Hepatica or Liverwort); *H. triloba* (Round-lobed H. or L.). Med. Prop.: Mucilaginous, astringent, pectoral, hepatic, tonic, deobstruent, diuretic.

Genus Anemone. (Wind-Flower). A. cylindrica (Longfruited Anemone); A. nemorosa (Wood A.); A. patens (Pasqueflower); A. Virginiana (Wind-bloom, Virginian A.). Med. prop.: Acrid, corrosive, rubefacient, emetic, cathartic, diuretic.

Genus Thalictrum. (Meadow Rue). T. dioicum (Early Meadow Rue); T. anemonoides (Rue Anemone, Poor-man's rhubarb). Med. prop.: Purgative, diuretic.

Genus Ranunculus. (Crowfoot, Buttercup). *R. acris* (Tall Crowfoot, Buttercups, Crowfoot-Buttercup); R. aquatilis (Common White Water-Crowfoot); R. abortivus (Small-flowered Crowfoot); R. Flammula (Smaller Spearwort); R. Pennsylvanicus (Bristly Crowfoot); R. repens (Creeping Crowfoot, Common C.); R. sceleratus (Marsh C., Cursed C.). Med. prop.: Very acrid, counter-irritant, vesicant, narcotic, emetic, antiscorbutic.

Genus Hydrastis. (Yellow Root). H. Canadensis (Yellow Puecoon, Golden-seal, Orange-root). Medical properties: Tonic, antiperiodic, laxative, cholagogue, detergent, alterative, diuretic, antiseptic.

Genus Actæa. (Baneberry). A. alba (White Cohosh, White Baneberry); A. spicata (Baneberry, Red Baneberry). Medical properties: Slightly acrid, purgative, emetic.

Genus Cimicifuga. (Bugbane). C. racemosa (Black Cohosh, Black Snake-root). Medical properties: Alterative, nervine, expectorant, emmenagogue, diuretic, anti-rheumatic, narcotic.

Genus Caltha. (Marsh Marigold). C. palustris (Marsh Marigold, erroneously called Cowslips). Medical properties: Mucilaginous, expectorant, pectoral.

Genus Aquilegia. (Columbine). A. Canadensis (Wild Columbine); *A. vulgaris* (Garden Columbine). Medical properties: Diuretic, emmenagogue, tonic, sudorific, discutient, diaphoretic, antiscorbutic.

Genus Delphinium. (Larkspur). *D. consolida* (Field Larkspur, Stagger-weed, Common Branching L.); D. exaltatum

(Tall L., Tall Wild L.). Medical properties: Diuretic, emmenagogue, vermifuge.

Order Magnoliaceæ. (Magnolia Family). 2 genera, 5 species:

Genus Liriodendron. (Tulip Tree). L. tulipfera (Tulip-tree, White-wood, Poplar, Yellow Poplar). Medical properties: Aromatic, bitter, tonic, stimulant, diaphoretic, antiperiodic, vermifuge.

Genus Magnolia. (Magnolia). *M. grandiflora, M. glanca,* M. acuminata (Cucumber-tree); M. umbrella vel M. tripetala (Umbrella-tree). Medical properties: Aromatic, bitter, tonic, stimulant, diaphoretic, astringent, antiperiodic, antirheumatic.

Order Anonaceæ. (Custard Apple Family). 1 genus, 1 species:

Genus Asimina. A. tribola (Common Papaw, Custard Apple.) Medical properties: Mucilaginous, anthelmintic.

Order Menispermaceæ. (Moonseed Family). 1 genus, 1 species:

Genus Menispermum. M. Canadense (Moonseed, Canadian M., Yellow Parilla). Medical properties: Tonic, bitter, narcotic, diuretic, stimulant, alterative, laxative.

Order Berberidaceæ. (Barberry Family). 4 genera, 4 species:

Genus *Berberis.* (Barberry). *B. vulgaris* (Common B.). Medical properties: Tonic, laxative, cathartic, antiperiodic, refrigerant, antiscorbutic.

Genus Caulophyllum. (Cohosh). C. thalictroides (Pappoose wood, Blueberry, Blue Cohosh, Cohosh, Blue or Yellow Ginseng, Squaw Root). Medical properties: Stimulant, emmenagogue, diaphoretic, diuretic, antispasmodic, parturient.

Genus Jeffersonia. (Twin Leaf). J. diphylla (Rheumatism-root, Helmet Pot, Ground Squirrel Pea). Medical properties: Antirheumatic, antisyphilitic, diuretic, alterative.

Genus Podophyllum. (Mandrake). P. peltatum (American Mandrake, May Apple). Medical properties: Cathartic, cholagogue, alterative, resolvent, emetic, diuretic, diaphoretic, emmenagogue, vermifuge.

Order Nymphæacheæ. (Water Lily Family). 4 genera, 4 species:

Genus Brasenia. (Water Shield). B. peltata (Little Water Lily, Frog-leaf, Water Target, Deerfoot). Medical properties: Mucilaginous, pectoral, astringent.

Genus Nelumbium. (Nelumbo. Sacred Bean). N. luteum (Yellow Nelumbo, Water Lily, Pond L., Water Shield, Water Nuts, Water Chinquepin, Sacred Bean). Medical properties: Refrigerant, laxative, emollient, diuretic, diaphoretic.

Genus Nymphæa. (Water Nymph, Water Lily). N. odorata (Sweet-scented Water Lily, White Pond Lily). Medical properties: astringent, discutient.

Genus Nuphar. (Yellow Pond Lily, Spotterdock). N. advena (Common Yellow Pond L.). Medical properties: Astringent, demulcent, emollient, discutient, emmenagogue.

Order Sarracenaceæ. (Pitcher Plant Family). 1 genus, 1 species:

Genus Sarracenia. (Side Saddle Flower). S. purpurea (Pitcher Plant, Huntsman's Cap, Side Saddle Flower). Medical properties: Bitter, astringent, tonic, stimulant, diuretic, laxative.

Order Papaveraceæ. (Poppy Family). 4 genera, 4 species:

Genus Papaver. (Poppy). *P. somniferum* (Common Poppy, Opium Poppy). Medical properties: Narcotic, anodyne, stimulant, hypnotic, relaxant.

Genus Stylophorum. (Claudine Poppy). S. diphyllum (Horn Poppy). Medical properties: Fœtid, narcotic, lithontryptic, diuretic.

Genus Argemone. (Prickly Poppy). A. Mexicana (Mexican Poppy). Medical properties: Anodyne, diuretic, cathartic, emetic.

Genus Sanguinaria. (Blood-root). S. Canadensis (Red-root, Red Puccoon-root, Indian Paint). Medical properties: Emetic, sedative, hepatic, tonic, stimulant, diuretic, febrifuge, alterative, emmenagogue.

Order Fumarraceæ. (Fumitory Family). 2 genera, 3 species:

Genus Dicentra, D. cucullaria (Dutchman's Breeches); D. Canadensis (Squirrel-corn, Colic-weed). Medical properties: Antisyphilitic, diuretic, alterative.

Genus Fumaria. (Fumitory). *F. officinalis* (Common Fumitory). Medical properties: Bitter, tonic, alterative, diuretic, laxative.

Order Cruciferæ. (Mustard Family). 10 genera, 15 species:

Genus Brassica. *B. alba* (White Mustard), B. nigra (Black Mustard). Medical properties: Stimulant, emetic, rubefacient.

Genus Sisymbrium. (Hedge Mustard). *S. officinalis* (Hedge Mustard). Medical properties: Detergent, antiscorbutic, lithontryptic.

Genus Nasturtium. (Water Cresss). *N. officinalis* (True Water Cress), N. palustre (Marsh Cress). Medical properties: Stimulant, depurative, antiscorbutic, deobstruent, diuretic, hepatic, emmenagogue.

*N. Armoracia* (Horse-radish) belongs to this genus and has the same properties, but it is more frequently used as a rubefacient and a condiment.

Genus Erysimum. (Treacle Mustard). E. cheirawthoides (Treacle M., Worm-seed M.). Medical properties: Stomachic, vermifuge.

Genus Barbarea. (Winter Cress). *B. vulgaris* (Common

Winter Cress), *B. præcox* (Scurvy-grass). Medical properties: Plant—antiscorbutic, pectoral, detergent; seed—acid, lithontryptic.

Genus Arabis. (Rock Cress). A. Canadensis (Sickle-pod). Medical properties: Diuretic, antiscorbutic.

Genus Cardamine. (Bitter Cress). C. rhomboidea (Spring Cress). Medical properties: Diuretic, antiscorbutic, stimulant, condiment.

Genus Camelina (False Flax). *C. sativa.* Medical properties: Oleaginous, vermifuge.

Genus Capsella (Shepherd's Pense). *C. Bursa-pastoris.* Medical properties: Detergent, astringent, acrid, rubefacient.

Genus Lepidium (Pepper-grass, Pepper-wort). L. campestre; L. Virginianum (Wild Pepper-grass). Medical properties: Leaves—pungent, diuretic, alterative, antiscorbutic; seed—acrid, astringent.

Order Capparidaceæ (Caper Family). 2 genera, 2 species:

Genus Polanisia. P. graveoleus (Wormwood, Stinkweed). Medical properties: Vesicant, counter-irritant.

Genus Gyandropsis *G. purtaphylla.* Medical properties: Similar to last.

Order Violaceæ (Violet Family). 1 genus, 7 species:

Genus Viola (Violet, Heartsease). V. cucullata (Common Blue Violet); V. pedata (Bird-foot Violet); V. blanda (Sweet White Violet, American Sweet V.); V. canina (Dog Violet); V. sagiltata (Arrow-leaved V., Rattlesnake's V.); *V. tricolor* (Pansy, Heartsease, Johnny Jumpup); V. arvenis. Medical properties: Mucilaginous, pectoral, emollient, alterative, laxtive, emetic, cathartic, discutient, vulnerary.

(*To be continued.*)

